# Seven Hub Genes Predict the Prognosis of Hepatocellular Carcinoma and the Corresponding Competitive Endogenous RNA Network

**DOI:** 10.1155/2022/3379330

**Published:** 2022-10-12

**Authors:** Xueqiong Han, Jianxun Lu, Chun Chen, Yongran Deng, Mingmei Pan, Qigeng Li, Huayun Wu, Zhenlong Li, Bingqiang Ni

**Affiliations:** ^1^Department of Oncology, The Fifth Affiliated Hospital of Guangxi Medical University & the First People's Hospital of Nanning, 89 Qixing Road, Nanning, Guangxi 530022, China; ^2^Department of Cardiology and Endocrinology, The Guangxi Zhuang Autonomous Region Workers' Hospital, Nanning, China

## Abstract

**Purpose:**

This study was aimed at identifying hub genes and ceRNA regulatory networks linked to prognosis in hepatocellular carcinoma (HCC) and to identify possible therapeutic targets.

**Methods:**

Differential expression analyses were performed to detect the differentially expressed genes (DEGs) in the four datasets (GSE76427, GSE6764, GSE62232, and TCGA). The intersected DEmRNAs were identified to explore biological significance by enrichment analysis. We built a competitive endogenous RNA (ceRNA) network of lncRNA-miRNA-mRNA. The mRNAs of the ceRNA network were used to perform Cox and Kaplan-Meier analyses to obtain prognosis-related genes, followed by the selection of genes with an area under the curve >0.8 to generate the random survival forest model and obtain feature genes. Furthermore, the feature genes were subjected to least absolute shrinkage and selection operator (LASSO) and univariate Cox analyses were used to identify the hub genes. Finally, the infiltration status of immune cells in the HCC samples was determined.

**Results:**

A total of 1923 intersected DEmRNAs were identified in four datasets and involved in cell cycle and carbon metabolism. ceRNA network was created using 10 lncRNAs, 67 miRNAs, and 1,923 mRNAs. LASSO regression model was performed to identify seven hub genes, SOCS2, MYOM2, FTCD, ADAMTSL2, TMEM106C, LARS, and KPNA2. Among them, TMEM106C, LARS, and KPNA2 had a poor prognosis. KPNA2 was considered a key gene base on LASSO and Cox analyses and involved in the ceRNA network. T helper 2 cells and T helper cells showed a higher degree of infiltration in HCC.

**Conclusion:**

The findings revealed seven hub genes implicated in HCC prognosis and immune infiltration. A corresponding ceRNA network may help reveal their potential regulatory mechanism.

## 1. Introduction

According to 2018 estimates provided by Bray about the incidence of cancer, liver cancer was responsible for 841,080 new cancer cases globally [1] and the incidence rates are expected to increase remarkably by the year 2030 [2]. Hepatocellular carcinoma (HCC) is the most prevalent form of liver cancer, representing 75-85% of all liver cancer cases [3]. Previous studies confirmed that the main pathogenic factors of HCC are the chronic hepatitis B virus (HBV) or hepatitis C virus (HCV) infection, alcoholic liver disease, and nonalcoholic fatty liver disease [4]. Statistics indicate that 30%-40% of HCC patients are diagnosed in the early stage [5]. Only a few methods for the prognosis and treatment of HCC, these methods have been limited because most HCC patients are diagnosed in advanced stages and surgically unresectable. Additionally, the methods depend mainly on the tumor stage [6] to reflect the development of tumor cells [7]. Therefore, identifying a sound risk stratification system can effectively treat and improve outcomes.

Competing endogenous RNA (ceRNA), including long noncoding RNA (lncRNA) and circle RNA (circRNA), can combine competitive microRNA (miRNA) and interfere with miRNA binding to messenger RNA (mRNA) to regulate gene expression and play important roles [8]. It has been shown that microRNAs, by virtue of their capacity to interact with many target genes, affect a wide variety of crucial biological processes, including growth, proliferation, and apoptosis of cells [9]. High expression of YKT6 [10] and MTFR2 [11] associated with progression and poor prognosis of HCC. Furthermore, EPHX2 was identified as an independent prognostic biomarker for overall survival of patients with HCC [12]. In summary, ceRNA is a factor that influences the incidence of HCC as well as its progression [13, 14]. The ceRNA was used to understand the interactions of complex genes and identified the potential biomarkers for diagnosing and treating HCC.

In this research, we mainly used the expression patterns of the databases in the Gene Expression Omnibus (GEO) (https://www.ncbi.nlm.nih.gov/geo/) and the Cancer Genome Atlas (TCGA) (https://portal.gdc.cancer.gov/) between HCC and normal samples to perform bioinformatics analysis. The purpose of this study is to construct a potentially competitive endogenous RNA (ceRNA) network to identify the underlying biological mechanisms of HCC. Furthermore, the model classifier and the risk score model were utilized to more precisely identify possible markers associated with prognosis in HCC patients.

## 2. Material and Methods

### 2.1. Data Preprocessing

There were a total of 600 HCC and 122 normal samples included within four different datasets (GSE76427, GSE6764, GSE62232, and TCGA datasets). RNA sequencing (RNA-seq) data, microRNA sequencing data, and the corresponding survival data of liver hepatocellular carcinoma (LIHC) patients were obtained from the TCGA database [15]. RNA-seq data and microRNA sequencing data of TCGA contained 369 primary HCC and 50 normal tissues as well as 370 primary HCC and 50 normal tissues, respectively. Corresponding clinicopathological features (age, sex, tumor differentiation degree, TNM stage, survival time, and status) were also obtained from the TCGA database are publicly available. Among these samples, one formalin sample and one relapse sample were excluded. Gene expression data of GSE76427, GSE6764, and GSE62232 were available from the GEO database. GSE76427 comprised 52 adjoining nontumor tissues as normal control and 115 tumor tissues with HCC. HCC patients included in this dataset had a mean age of 63.45 ± 12.63 years, and 93 male and 22 female [16], conducted by GPL10558 platform (Illumina HumanHT-12 V4.0 expression beadchip). GSE6764 was conducted by the GPL570 platform (Affymetrix Human Genome U133 Plus 2.0 Array) and comprised 35 HCC tissues and 10 adjoining nontumor tissues. HCV infection cases observed in 13 samples from cirrhotic tissues and 17 samples from dysplastic nodules were excluded [17]. GSE62232 containing 81 solid HCC and 10 nontumor liver samples was acquired on the basis of the GPL570 platform (Affymetrix Human Genome U133 Plus 2.0 Array). The individuals of HCC included in this dataset had a mean age of 60.6 ± 13.49 years, and 67 male and 14 female [18]. The “varianceStabilizingTransformation” function of the DESeq2 package [19] was utilized for normalizing the expression patterns of RNA sequencing data and microRNA sequencing from the TCGA dataset. Furthermore, expression profiles of GSE6764 and GSE62232 were normalized using the “RMA” function in the Affy package. The expression profile of GSE76427 was used to normalize by the “lumiExpresso” function in the Lumi R package.

### 2.2. Identification of Differentially Expressed Genes

By employing the limma package in R, differential expression analysis was carried out for gene expression to find the DEGs between HCC tumor tissues and nontumor liver tissues in GSE76427, GSE6764, and GSE62232 [20]. We also utilized the DESeq2 package [19] to identify the DEGs of TCGA. DEGs of all the four datasets (GSE76427, GSE6764, GSE62232, and TCGA) were deemed to have statistical significance if the adjusted *P* value was <0.05. Subsequently, the intersected DEGs of four datasets were identified using the Venn diagrams to obtain consistent expression, including upregulated and downregulated DEGs.

### 2.3. Gene Ontology (GO) and Pathway Enrichment Analysis

Cellular components (CCs), Biological processes (BPs), and molecular functions (MFs) of intersected DEGs were used to explore the biological significance by performing a GO analysis. Kyoto Encyclopedia of Genes and Genomes (KEGG) was utilized to explore significantly altered pathways enriched in the gene list. GO and KEGG pathways were executed with the help of the clusterProfile package [21]. Gene enrichment in the GO and KEGG pathway with *P* value <0.05 were judged as significant.

### 2.4. Gene Set Enrichment Analysis (GSEA)

GSEA is a computational approach that enables gene sets to identify genomes excessively increasing or decreasing between biological phenotypes [22]. GSEA analysis was carried out to ascertain the functions premised on the expressed profiles of TCGA using the GSEA software (Version 4.1.0).

### 2.5. ceRNA Network Analysis

We examined the regulated miRNAs using up/downregulated lncRNAs in four datasets by Starbase databases (http://starbase.sysu.edu.cn/index.php) while retaining the opposite expression direction of lncRNAs and miRNAs. Among them, we extracted the intersection of the regulated miRNAs of TCGA for the next analysis. Meanwhile, we screened for miRNA-regulated mRNAs on the Targetscan databases (https://www.targetscan.org/vert_72/) while retaining the same expression direction of lncRNAs and mRNAs. Subsequently, we generated the lncRNA-miRNA-mRNA regulatory network by downloading the binding sites of mRNA, miRNA, and lncRNA using the Starbase and Targetscan databases.

### 2.6. Establishment of Random Survival Forest Model and Least Absolute Shrinkage and Selection Operator (LASSO) Regression Model

The mRNAs of the ceRNA regulatory network were used to perform Cox and Kaplan-Meier analyses in TCGA to obtain prognosis-related genes. We selected the prognosis-related genes for the area under the curve (AUC) analysis by pROC package. Next, we used prognosis-related genes with an AUC >0.8 to construct the random survival forest model with the help of the RandomSurvivalForest R package. It was determined that genes with a relative importance >0.4 were the ultimate feature genes by examining the link between the error rate and the number of classification trees. Moreover, the feature genes were used to construct the LASSO model and the hub genes were obtained using “cv.glmnet” function in glmnet R package [23]. Subsequently, a univariate Cox analysis was carried out on the hub genes to determine the prognostic significance utilizing the forestplot R package. The hub genes with a hazard ratio >1 were considered to lead to a poor prognosis.

### 2.7. Seven Hub Gene-Risk Scores Based on Cox Regression Analysis

After performing a multivariate Cox regression analysis premised on the outcomes of seven hub genes, risk scores were then computed. Following the construction of the risk score model premised on the median risk score, the HCC patients of the TCGA dataset were further classified into high- and low-risk groups, and their overall survival (OS) rates were compared. Furthermore, to determine the impact that hub genes have on the HCC patients' prognoses, a nomogram was developed with the use of the rms package of the R program. With the help of the ggstatsplot package, correlations between hub genes and risk scores were derived.

### 2.8. Identification of Key Gene

To further illustrate the expression of hub genes between HCC and control samples in the TCGA dataset, we created a heat map and a violin plot. Above all, time-dependent receiver operating characteristic curve (ROC) analysis was conducted using the “survivalROC” R package to evaluate the prediction accuracy of the 1-, 3-, 5- year of the key gene. The Kaplan–Meier survival curve was utilized to make a comparison between the high- and low-risk groups for the survival of the key gene. Furthermore, *P* adjust value <0.05 was selected as the criterion for determining differentially expressed miRNAs (DEmiRs), after which we plotted the associated ceRNA regulatory network of key genes.

### 2.9. Immune Infiltration Analysis

Single-sample gene set enrichment analysis (ssGSEA) calculated the degree of immune cell infiltration in the HCC patients of 24 immune cell types using marker gene sets [24]. Using the limma R program, variations in the types of immune cells seen between HCC and control samples were computed Radar and scatter plots show correlation plots of risk score of immune cells and hub gene. Second, we adopted the Pearson correlation to determine the link between the seven hub genes and the immune cell types. Additionally, The CIBERSORT algorithm was utilized to execute an analysis of the infiltration levels of 22 different types of immune cells in HCC samples taken from the TCGA dataset.

### 2.10. Statistical Analysis

The analyses of the present study were conducted utilizing the Bioinforcloud platform (http://www.bioinforcloud.org.cn).

## 3. Results

**Figure 1:**
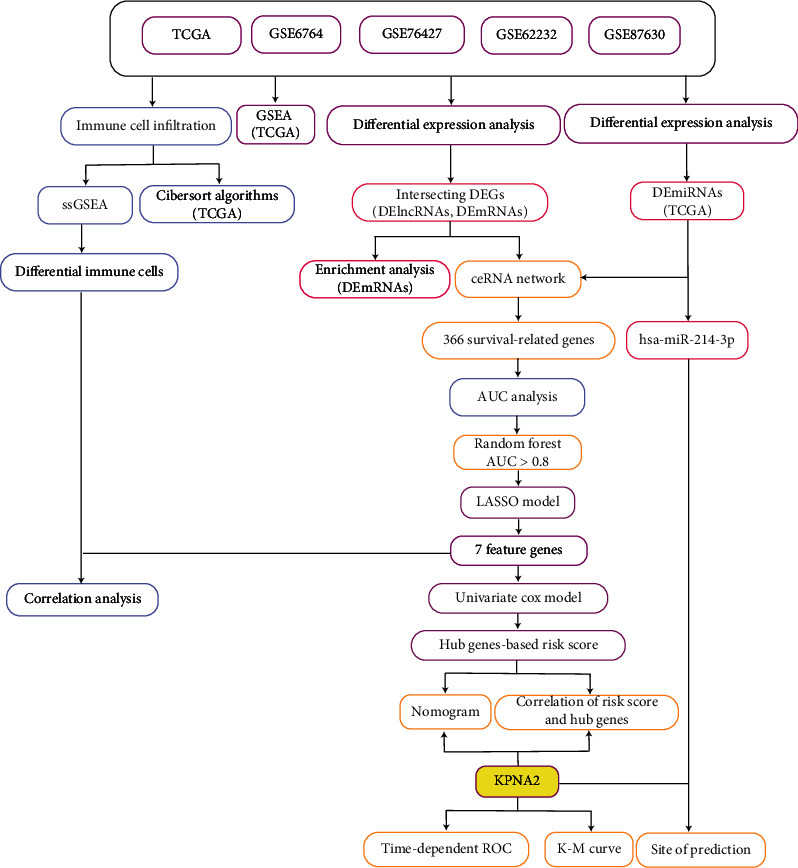
The detailed flow chart for this research AUC, area under the curve; DElncRNA, differentially expressed long noncoding RNA; DEG, differentially expressed genes; DEmRNA, differentially expressed messenger RNA; Gene Set Enrichment Analysis; LASSO, least absolute shrinkage, and selection operator; K-M curve, Kaplan-Meier curve; TCGA, The Cancer Genome Atlas; ssGSEA, single sample Gene Set Enrichment Analysis; ROC, receiver operating characteristic curve.

Workflow of the present study (Figure 1).

### 3.1. Biological Function of DEGs between HCC and Controls

**Figure 2:**
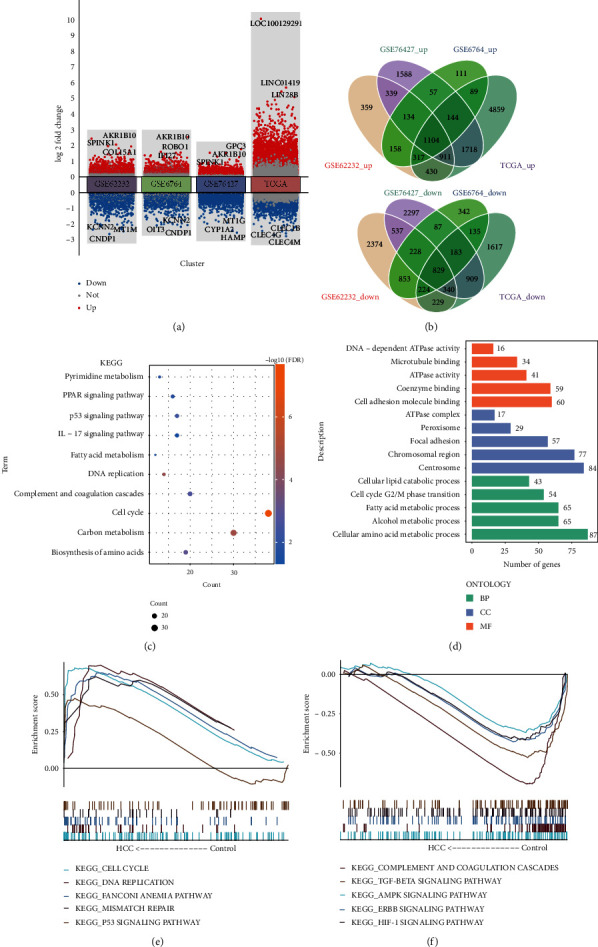
Enrichment analysis of differentially expressed genes (DEGs). (a) DEGs of GSE62232, GSE6764, GSE76427, and TCGA datasets between HCC and controls. Red denotes upmodulated DEGs, whereas blue denotes downmodulated DEGs. (b) DEGs in the same direction across all four datasets are shown by a Venn diagram. (c) Intersected DEGs of four datasets were involved in pathways by enrichment analysis. (d) Intersected DEGs of four datasets were involved in biological process, molecular function, and cellular component. (e, f) Gene set enrichment analysis illustrated the head and tail of five pathways enriched in HCC patients. KEGG, Kyoto Encyclopedia of Genes and Genomes; HCC, hepatocellular carcinoma; CC, cellular component; MF, molecular function; BP, biological process.

To obtain dysfunctional genes associated with HCC, we identified DEGs between HCC and controls (Figure 2(a)). In total, 9,366 DEGs were identified in GSE62232, 11405 DEGs in GSE76427, 4995 DEGs in GSE7696, and 14,038 DEGs in TCGA. A total of 1,933 intersected DEmRNAs were detected in four datasets, including 10 lncRNA and 1,923 mRNA. Among them, 1,104 were upregulated and 829 downregulated DEGs in HCC and controls (Figure 2(b)). Intersected DEGs were involved in the cell cycle and carbon metabolism (Figure 2(c)). Additionally, intersected DEGs were involved in 1,584 BP, 210 CC, and 213 MF (Figure 2(d)). GSEA showed that genes of the TCGA dataset were positively linked to DNA replication and the cell cycle (Figure 2(e)) and were inversely linked to the ERBB signaling pathway and HIF-1 signaling pathway (Figure 2(f)).

### 3.2. Identification of Hub Genes Related to Prognosis

**Figure 3:**
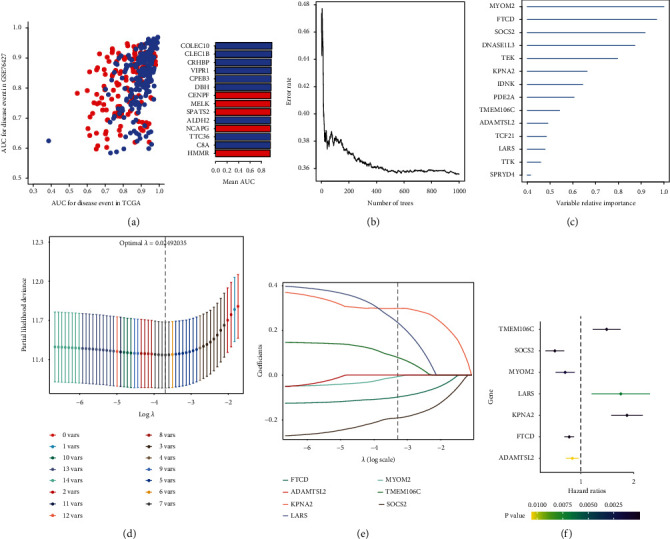
Identification of hub genes by least absolute shrinkage and selection operator (LASSO) and univariate Cox models. (a) The top 14 genes are shown with AUC>0.80 in TCGA and GSE76427. Genes that have been upregulated are shown in red, whereas genes that have been downregulated are shown in blue. (b) The correlation between the number of trees and the error rate. (c) A random forest model was constructed to determine the importance of the 14 genes in an order. (d) LASSO coefficient profiles of the 7 feature genes. (e) 10-fold cross-validation of parameter selection in the LASSO analysis. (f) Univariate analysis of feature genes. The feature genes with hazard ratios >1 are poor prognosis genes, and hazard ratios <1 are protective genes. TCGA, The Cancer Genome Atlas; AUC, area under the curve.

The ceRNA regulatory network was constructed using the 366 prognosis-related mRNAs that were obtained from Kaplan-Meier and Cox survival analyses (Table S1). Based on AUC analysis, 366 prognosis-related mRNAs were analyzed to determine their possible involvement in the GSE76427 and TCGA datasets. As depicted in Figure 3(a), 211 genes with an AUC of <0.80 were found in both datasets. Figures 3(b) and 3(c) provide an orderly representation of the association between the error rate for the data, the number of classification trees, and the importance of the 14 genes. LASSO regression model was performed to identify 7 hub genes, SOCS2, MYOM2, FTCD, ADAMTSL2, TMEM106C, LARS, and KPNA2 (Figures 3(e) and 3(f)). In univariate Cox regression analysis, TMEM106C, LARS, and KPNA2 had a poor prognosis (Figure 3(f)).

### 3.3. Construction of Hub Genes and Calculation of Risk Score in HCC

**Figure 4:**
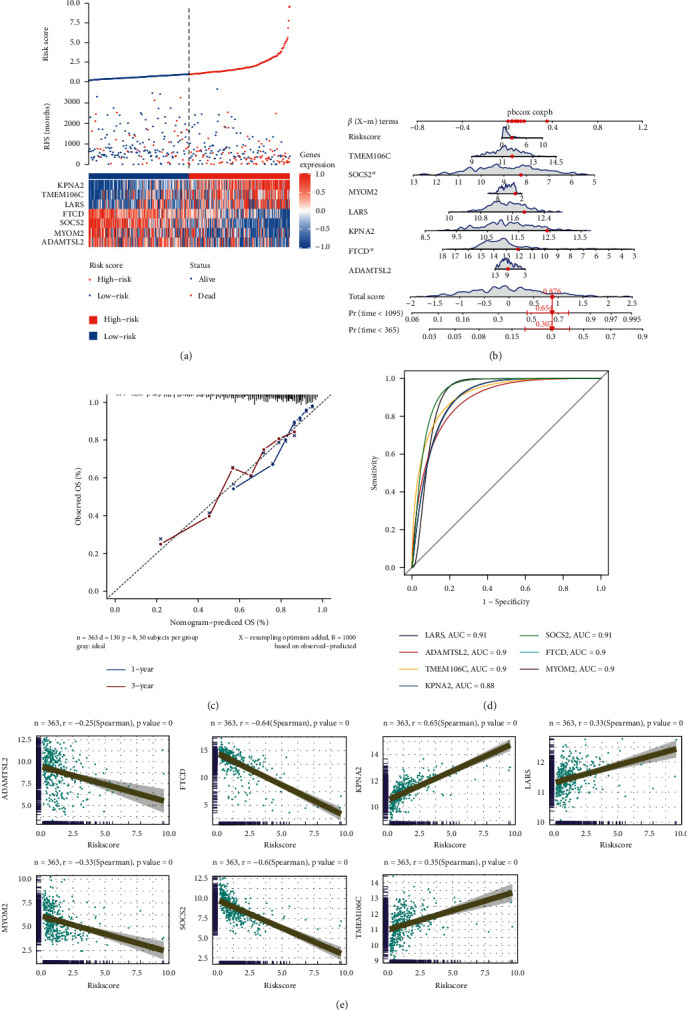
Seven feature gene-risk scores based on Cox analyses. (a) Expression, risk score, and survival status of seven genes in HCC patients of the TCGA dataset. (b) Nomogram for the prediction of 1- and 3-year overall survival (OS) for HCC patients with seven feature genes. (c) Calibration curves for seven feature genes and 1-, 3-year OS in the validation set (TCGA). (d) AUCs value of seven feature genes. (e) Correlation of risk score and seven hub genes. OS, overall survival; AUC, area under the curve.

The distributions of the risk scores, RFS and hub gene expression of the 369 patients in the TCGA dataset are shown in Figure 4(a). The hub genes were incorporated into a nomogram model to predict the HCC patients' prognoses (Figure 4(b)). The calibration curve demonstrated excellent agreement between the observed and predicted OS over 1 and 3 years in the TCGA cohort (Figure 4(c)). The AUC analysis of seven hub genes as illustrated in Figure 4(d) and the relevant findings illustrated that the seven hub genes had a better diagnostic power in the prognostic model. We additionally examined the link between hub genes and risk scores and found that KPNA2, LARS, and TMEM106C had a positive link to risk scores, whereas an inverse correlation was observed between ADAMTSL2, MYOM2, FTCD, SOCS2, and risk scores (Figure 4(e)).

### 3.4. KPNA2 as a Key Gene in Prognosis for HCC

**Figure 5:**
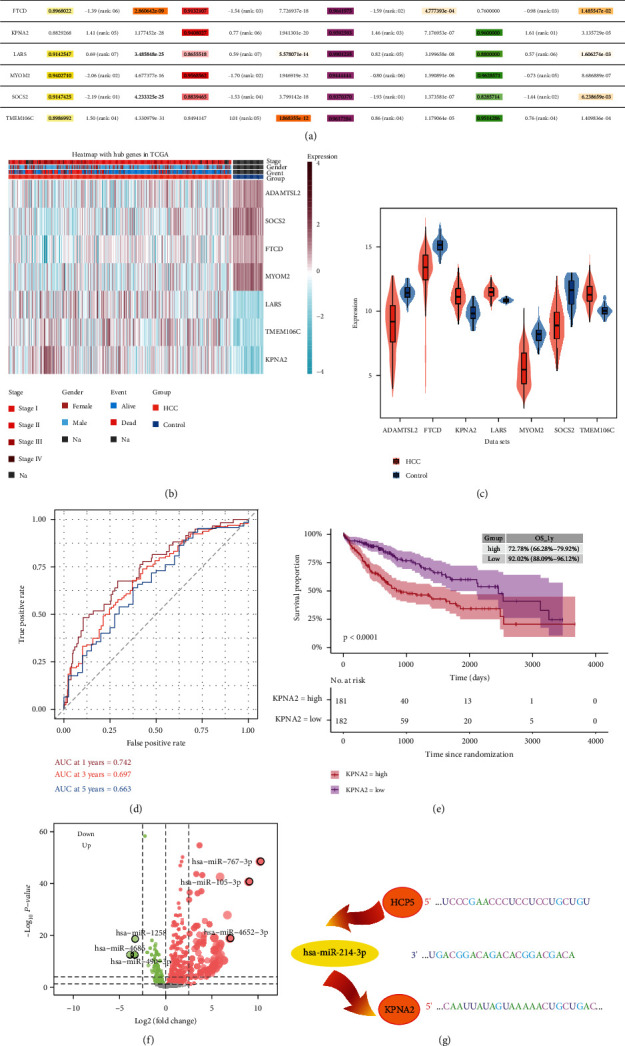
Expression of hub genes and construction of ceRNA regulated network. (a) The log-fold change, AUC, and adjusted *P* value of seven genes in TCGA, GSE76427, GSE62232, and GSE6764. (b) Stage, gender, event, and expressed levels were shown in HCC and control in HCC. (c) Violin plots illustrated the expression of seven hub genes. The thick black bar in the middle indicates the interquartile range, and the black line extending from it represents the 95% confidence interval. (d) Time-dependent receiver operating characteristic curve analysis displayed the AUC values over 1, 3, and 5 years. (e) Estimating the survival time of the KPNA2d by Kaplan-Meier survival curve. (f) Differentially expressed miRNAs were identified between controls and HCC samples in TCGA. Red represents upregulated miRNAs, whereas green represents downregulated miRNAs. (g) Bind sites of HCP5/has-miR-214-3p/KPNA2. Orange indicates upregulated, and yellow indicates down-regulated. HCC, hepatocellular carcinoma; AUC, area under the curve; TCGA, The Cancer Genome Atlas.

In four datasets (GSE76427, GSE6764, GSE62232, and TCGA), the results suggested that hub genes of AUCs value, adjusted *P* value, and fold change were depicted in Figure 5(a). A heat map showed the expression level of hub genes, stage, gender, event, and groups in TCGA (Figure 5(b)). In comparison with controls, ADAMTSL2, FTCD, KPNA2, LARS, and TMEM106C were found to be expressed at a high level in HCC (Figure 5(c)). Time-dependent ROC survival analysis was employed to examine the prognosis of KPNA2 and the findings illustrated that the AUCs values over 1, 3, and 5 years were all greater than 0.66 (Figure 5(d)). Moreover, the predicted 1-year survival time for HCC patients indicated that KPNA2 had considerably improved OS (Figure 5(e)). The volcano plot showed a total of 901 DEmiRs, comprising 730 upmodulated miRNAs and 171 downmodulated miRNAs (Figure 5(f)). Ultimately, Figure 5(g) depicts the obtained binding sites within the lncRNA HCP5-KPNA2-miR-214-3p as ceRNA regulatory network. Above all, KPNA2 performs an essential function in the ceRNA regulatory network, making it a key gene in the prognosis and fundamental biological processes involving HCC.

### 3.5. Estimation of Infiltrating Immune Cells in HCC

**Figure 6:**
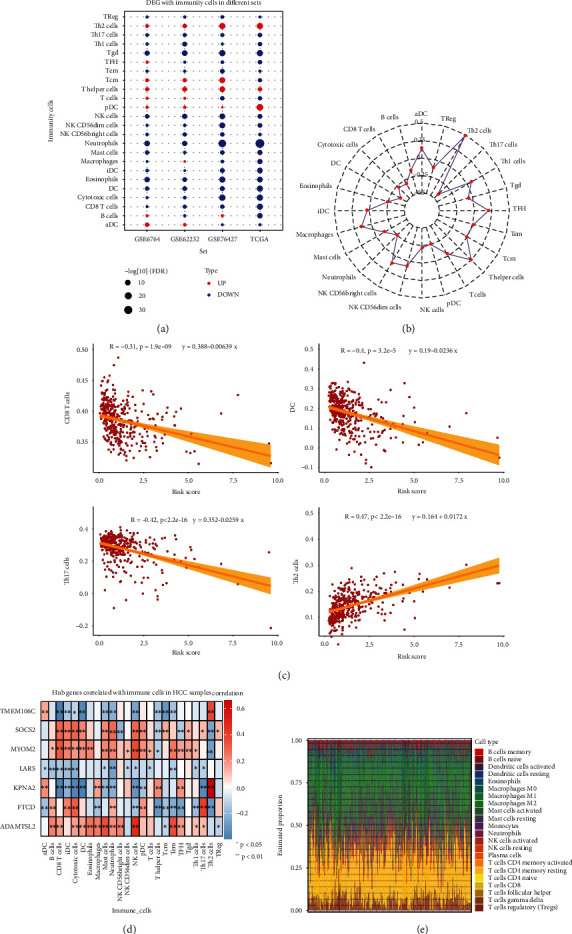
Immune cell infiltration in HCC. (a) Single sample gene set enrichment analysis was used to estimate the infiltration of immune cells. Red indicates high infiltration and blue indicates low infiltration. (b) Radar plot shows the correlation between 24 immune cell types and risk score of seven hub genes. (c) Correlation scatter plots shows the most significant infiltrating cells and risk score. (d) Correlation with seven hub genes and immune cells in HCC samples. (e) Estimated proportions of 22 immune cell types. DEGs, differentially expressed genes; HCC, hepatocellular carcinoma.

T helper (Th) 2 cells, T helper cells, and plasmacytoid dendritic cells (pDCs) all have higher degrees of infiltration between HCC and controls in four datasets (Figure 6(a)).  Figure 6(b) depicts the association between the median risk score and the types of immune cells. Among them, CD8 T cells, Th17 cells, and DC exhibited a substantially positive correlation, whereas Th2 cells exhibited a significantly negative correlation (Figure 6(c)). By determining the Pearson correlation between the hub genes and the 24 different types of immune cells, we observed that KPNA2 and Th2 cells had a considerably high association in HCC samples (Figure 6(d)). To further evaluate the proportion of immune cells for TCGA, the findings indicated that HCC samples were extensively infiltrated by Macrophages M2 (Figure 6(e)).

## 4. Discussion

HCC is a malignancy with a high death rate and an unfavorable prognosis, necessitating novel diagnostic and therapeutic markers [25]. In this research, we determined the seven hub genes of HCC prognosis as vital biomarkers, which can be used to improve outcomes of patients with HCC. We constructed a ceRNA regulatory network to explore the biological mechanism, including lncRNA HCP5-hsa-miR-214-3p-KPNA2. Furthermore, we also found a high degree of immune cell infiltration with seven hub genes.

Intersected DEGs of four datasets were involved in the cell cycle and carbon metabolism. Previous research has shown that cell cycle regulation inhibits the proliferative ability of HCC cells [26]. Importantly, GSEA showed that genes of the TCGA dataset were enriched in the cell cycle and carbon metabolism. Studies have shown that tumor cells often regulate the genes of the cell cycle producing damage and inactivation of this pathway may be involved in tumor development [27]. Furthermore, high expression of miR-452-5p performs an integral function in the progression of HCC through carbon metabolism [28].

Seven hub genes (SOCS2, MYOM2, FTCD, ADAMTSL2, TMEM106C, LARS, and KPNA2) involved in the process of the HCC prognosis were identified. SOCS2 was associated with distinct stages that indicated poor survival outcomes for patients with HCC [29]. Previous studies have shown that FTCD is a protective factor in HCC development and prognosis [30]. Overexpression of TMEM106C [31] and KPNA2 [32] predicted an unsatisfactory prognosis in HCC patients. Downregulated MYOM2 was observed in a majority of clinical cases of breast cancer [33], however, the role of MYOM2, ADAMTSL2, and LARS in HCC development and prognosis remains unclear.

In this present research, in the TCGA liver cancer cohorts, a high level of KPNA2 expression accurately predicted the 1-, 3-, and 5-year survival times, with AUCs of 0.742, 0.697, and 0.663, correspondingly. A risk score and nomogram model also indicated that high-expressed KPNA2 led to an unfavorable prognosis. There was also a strong association between the risk score and KPNA2, which suggests that KPNA2 could be a crucial biological marker in determining the prognosis of patients with HCC. Recently, an increasing number of studies have demonstrated that lncRNA and miRNA primarily mediated posttranscriptional regulation, and that dysregulation of this process has been linked to many malignancies [34]. Interestingly, through targeting KPNA2, the lncRNA HCP5 served as a ceRNA, which had the effect of adversely modulating the expression of miR-214-3p. Downmodulation of lncRNA HCP5 in HCC tissues, when contrasted to normal samples, could affect the proliferation, metastatic and invasive, while the relevant mechanism of HCC still needs to be elucidated [35]. miR-214-3p is shown to modulate cell growth, metastasis, and apoptosis in HCC cells, endometrial cancer cells, and retinoblastoma cells by directly targeting certain genes linc00665 [36], ATWIST1 [37], BCB1, and XIAP [38]. In general, this ceRNA regulatory network further helps us understand the regulatory mechanisms of these genes in HCC.

ssGSEA indicated that Th2 cells and plasmacytoid dendritic cells (pDCs) all exhibited a higher degree of infiltration between HCC and controls. An intratumoral infiltration of pDCs is predictive of an unfavorable prognosis among patients who undergo curative resection for HCC; pDCs exist in numerous primary as well as metastatic human neoplasms [39]. Furthermore, the significantly positive correlation between Th2 cells and KPNA2 that Th2 cells were linked to HCC patient survival [40]. Th17 cells, dendritic cells (DCs), and CD8 T cells were positively linked to the risk score of prognosis. The levels of Th17 cells were substantially elevated in tumors of patients with HCC [41]. DCs and CD8+ T cells have increased infiltration levels and are associated with relapse, compared with primary HCC [42]. The seven hub genes were defined as biomarkers for OS and we constructed a high immune cell infiltration model to predict the HCC patients' prognoses.

Despite the new findings at the level of bioinformatics analysis, understanding of the prognosis and immune-related biomarkers are still limited. Firstly, the markers associated with HCC currently lack sufficient sensitivity and specificity. Secondly, molecular and animal experiments are needed to verify the biomarkers and apply the biomarkers from preclinical studies in clinical practice.

## 5. Conclusion

SOCS2, MYOM2, FTCD, ADAMTSL2, TMEM106C, LARS, and KPNA2 are vital biomarkers and involved in the process of the HCC prognosis and immune infiltration.

## Data Availability

The datasets (GSE76427, GSE6764, GSE62232) supporting the conclusions of this article are available in the Gene Expression Omnibus (http://www.ncbi.nlm.nih.gov/geo) and the Cancer Genome Atlas https://portal.gdc.cancer.gov/) database.

## Supplementary Materials

Supplementary Materials Supplementary Table 1 366 prognosis-related mRNA list.
