# Supplementary material for: Seven Hub Genes Predict the Prognosis of Hepatocellular Carcinoma and the Corresponding Competitive Endogenous RNA Network
Source: J Oncol. 2022 Oct 12;2022:3379330. doi: 10.1155/2022/3379330 (PMC9581604; doi:10.1155/2022/3379330)
Supplement: Supplementary Materials — Supplementary Table 1 366 prognosis-related mRNA list. [file 3379330.f1.docx]

**Supplementary**

**Table 1.** 366 prognosis-related mRNA by Kaplan-Meier and Cox survival analysis

| node | type | logFC | node | type | logFC |
| --- | --- | --- | --- | --- | --- |
| DHRS4-AS1 | lncRNA | -0.952915032 | GNE | mRNA | -1.577997589 |
| LINC01018 | lncRNA | -1.479675587 | GNMT | mRNA | -1.541084888 |
| DIO3OS | lncRNA | -1.156506994 | GPD1L | mRNA | 0.808271787 |
| ZEB1-AS1 | lncRNA | 0.847344185 | GPT | mRNA | -0.814347738 |
| CRNDE | lncRNA | 3.345472996 | GRAMD1C | mRNA | -1.593691927 |
| HCP5 | lncRNA | 0.768170427 | GTPBP4 | mRNA | 0.469181538 |
| hsa-miR-545-5p | micRNA | 0.436178647 | GYS2 | mRNA | -2.180036101 |
| hsa-miR-584-5p | micRNA | 0.488272568 | H2AFX | mRNA | 1.445378781 |
| hsa-miR-1276 | micRNA | 1.833566279 | H2AFY | mRNA | 0.649190555 |
| hsa-miR-522-3p | micRNA | 5.316233276 | HAAO | mRNA | -1.366649154 |
| hsa-miR-499a-5p | micRNA | 0.876658155 | HAO1 | mRNA | -1.456609446 |
| hsa-miR-942-5p | micRNA | 0.90672763 | HAO2 | mRNA | -2.356889219 |
| hsa-miR-340-5p | micRNA | 0.24059149 | HDAC1 | mRNA | 0.353618139 |
| hsa-miR-3681-5p | micRNA | 1.394706011 | HELLS | mRNA | 2.403273191 |
| hsa-miR-525-5p | micRNA | 4.98610964 | HGFAC | mRNA | -1.759395017 |
| hsa-miR-660-5p | micRNA | 1.308953786 | HJURP | mRNA | 3.891344749 |
| hsa-miR-629-5p | micRNA | 0.501132689 | HMGCL | mRNA | -1.282904246 |
| hsa-miR-4677-3p | micRNA | 0.937650289 | HMMR | mRNA | 3.227629749 |
| hsa-miR-524-5p | micRNA | 4.524110732 | HOGA1 | mRNA | -1.813506638 |
| hsa-miR-142-5p | micRNA | -0.854914041 | HPX | mRNA | -1.729095244 |
| hsa-miR-625-5p | micRNA | 0.819012024 | IDNK | mRNA | -1.17646189 |
| hsa-miR-545-3p | micRNA | 0.436178647 | IFNAR1 | mRNA | -0.790383378 |
| hsa-miR-6720-5p | micRNA | 2.25847253 | IGF1 | mRNA | -1.300854948 |
| hsa-miR-182-5p | micRNA | 3.453459727 | IGF2BP3 | mRNA | 4.745849359 |
| hsa-miR-218-5p | micRNA | 0.365620417 | IGFALS | mRNA | -3.171260414 |
| hsa-miR-324-3p | micRNA | 0.768584154 | IGFBP4 | mRNA | -1.045476712 |
| hsa-miR-202-5p | micRNA | 1.004330514 | IL33 | mRNA | -1.656850982 |
| hsa-miR-301a-3p | micRNA | 1.316405245 | INMT | mRNA | -2.444993628 |
| hsa-miR-541-5p | micRNA | 2.681724648 | ITGAD | mRNA | -1.060331941 |
| hsa-miR-330-3p | micRNA | 0.837256797 | ITGAV | mRNA | 0.677948059 |
| hsa-miR-126-5p | micRNA | -0.272360626 | ITGB1BP1 | mRNA | 0.883317602 |
| hsa-miR-204-5p | micRNA | 1.184161006 | ITIH1 | mRNA | -1.089276852 |
| hsa-miR-362-5p | micRNA | 1.070803944 | IVD | mRNA | -1.219345516 |
| hsa-miR-485-5p | micRNA | 1.183081754 | IYD | mRNA | -1.614132038 |
| hsa-miR-130b-3p | micRNA | 1.036286232 | KBTBD11 | mRNA | -2.344441375 |
| hsa-miR-423-5p | micRNA | 0.760048008 | KDM8 | mRNA | -2.422140489 |
| hsa-miR-214-3p | micRNA | -1.025559346 | KIF14 | mRNA | 3.089060128 |
| hsa-miR-493-3p | micRNA | 1.299885024 | KIF15 | mRNA | 3.348416233 |
| hsa-miR-211-5p | micRNA | 1.05830488 | KIF23 | mRNA | 3.287151547 |
| hsa-miR-29a-3p | micRNA | -0.40459568 | KIF3A | mRNA | 0.671685908 |
| hsa-miR-409-3p | micRNA | 1.036373427 | KNOP1 | mRNA | 0.753391799 |
| hsa-miR-222-3p | micRNA | 1.416745024 | KNSTRN | mRNA | 0.96673933 |
| hsa-miR-532-5p | micRNA | 1.395631089 | KNTC1 | mRNA | 1.92551563 |
| hsa-miR-206 | micRNA | 0.71052004 | KPNA2 | mRNA | 1.410618986 |
| hsa-miR-3064-5p | micRNA | 0.490522043 | LARP4B | mRNA | 0.399408804 |
| hsa-miR-301b-3p | micRNA | 1.89512369 | LARS | mRNA | 0.691241314 |
| hsa-miR-221-3p | micRNA | 1.581089245 | LDB2 | mRNA | -0.867752689 |
| hsa-miR-324-5p | micRNA | 0.768584154 | LDHD | mRNA | -1.315410745 |
| hsa-miR-140-5p | micRNA | 0.83602857 | LILRB5 | mRNA | -1.924735558 |
| hsa-miR-518e-5p | micRNA | 5.232869494 | LIN9 | mRNA | 1.547718739 |
| hsa-miR-27b-3p | micRNA | -0.461886468 | LPA | mRNA | -2.327283494 |
| hsa-miR-486-5p | micRNA | -0.684468796 | LRRC3 | mRNA | -0.757587149 |
| hsa-miR-133b | micRNA | -0.96452174 | LRRC42 | mRNA | 0.521285888 |
| ABAT | mRNA | -1.612981349 | LTK | mRNA | -1.427842156 |
| ABCA8 | mRNA | -1.488806715 | MAD2L1 | mRNA | 2.149948437 |
| ABCC5 | mRNA | 1.087609315 | MARCKS | mRNA | 0.669265894 |
| ACADS | mRNA | -1.782849468 | MASP1 | mRNA | -1.617125978 |
| ACADSB | mRNA | -1.770621026 | MASTL | mRNA | 0.555173087 |
| ACAT1 | mRNA | -1.400519349 | MAT1A | mRNA | -1.816525262 |
| ACO1 | mRNA | -0.724085547 | MCM2 | mRNA | 2.395568171 |
| ACSL1 | mRNA | -1.843866511 | MCM4 | mRNA | 1.618374792 |
| ADAMTSL2 | mRNA | -1.514666489 | MCM7 | mRNA | 1.487033549 |
| ADH4 | mRNA | -2.371422946 | MELK | mRNA | 3.786596648 |
| ADH6 | mRNA | -1.533516457 | MGMT | mRNA | -0.716099617 |
| ADI1 | mRNA | -0.950115393 | MICU1 | mRNA | -0.573937154 |
| ADK | mRNA | -1.113564416 | MMAA | mRNA | -1.181388873 |
| ADRA1A | mRNA | -3.114771077 | MOGAT2 | mRNA | -2.221646779 |
| ADRA2B | mRNA | -2.233931014 | MPDZ | mRNA | -1.121410875 |
| AKR1B10 | mRNA | 3.634161491 | MRGBP | mRNA | 0.894817515 |
| AKR1D1 | mRNA | -1.911893553 | MROH8 | mRNA | -0.75043103 |
| ALDH2 | mRNA | -1.682143196 | MRPL54 | mRNA | -0.319254397 |
| ALDH6A1 | mRNA | -1.75379666 | MSH2 | mRNA | 1.288939534 |
| ALDOA | mRNA | 1.121978368 | MSRA | mRNA | -1.38346479 |
| ALDOB | mRNA | -1.550816479 | MYOM2 | mRNA | -2.058325928 |
| ALPL | mRNA | -1.564889789 | MZT1 | mRNA | 0.787100098 |
| AMDHD1 | mRNA | -1.372090416 | N4BP2L1 | mRNA | -1.455302236 |
| ANKRD27 | mRNA | 0.936767216 | NAA40 | mRNA | 1.081067362 |
| ANLN | mRNA | 3.62139145 | NAP1L1 | mRNA | 0.603346562 |
| APBA1 | mRNA | -0.522698042 | NARS | mRNA | 0.299758658 |
| APOC3 | mRNA | -1.178847347 | NAT10 | mRNA | 0.364018114 |
| ARHGEF26 | mRNA | -1.291083761 | NCAPG | mRNA | 3.796181025 |
| ASPA | mRNA | -1.9317693 | NCAPG2 | mRNA | 1.229693935 |
| AZGP1 | mRNA | -1.56885559 | NDRG2 | mRNA | -1.509992994 |
| BAIAP2 | mRNA | -0.401628963 | NDRG3 | mRNA | 0.985171361 |
| BARD1 | mRNA | 1.259571743 | NEDD1 | mRNA | 0.363158736 |
| BCAT1 | mRNA | 1.411387483 | NEIL3 | mRNA | 4.159087509 |
| BCO2 | mRNA | -3.108736544 | NOSTRIN | mRNA | -0.382586298 |
| BEND2 | mRNA | -1.570286959 | NR1I2 | mRNA | -1.367951987 |
| BRIX1 | mRNA | 0.43689496 | NT5C3A | mRNA | 0.461867849 |
| BTBD10 | mRNA | 0.265616433 | NTF3 | mRNA | -3.486910123 |
| BUB1B | mRNA | 3.506266301 | NUDT1 | mRNA | 1.834703071 |
| C11orf54 | mRNA | -1.169882029 | NUF2 | mRNA | 4.197561355 |
| C1RL | mRNA | -1.650476765 | NUP155 | mRNA | 0.719740644 |
| C5orf34 | mRNA | 2.402282027 | NUP205 | mRNA | 0.605952107 |
| C6 | mRNA | -1.955005402 | NUP43 | mRNA | 0.551569055 |
| C7 | mRNA | -2.037362678 | P2RY8 | mRNA | 0.881785001 |
| C8A | mRNA | -1.830987459 | PA2G4 | mRNA | 0.492384688 |
| CAMSAP2 | mRNA | 0.591551054 | PAK1IP1 | mRNA | 0.315243262 |
| CAT | mRNA | -1.428706296 | PARPBP | mRNA | 2.695701213 |
| CBFA2T3 | mRNA | -1.948331257 | PBLD | mRNA | -1.766864608 |
| CBLN3 | mRNA | -0.847380016 | PCK1 | mRNA | -2.230102545 |
| CBS | mRNA | -0.919050122 | PCSK6 | mRNA | -0.837472241 |
| CC2D1B | mRNA | 0.408356136 | PDE2A | mRNA | -1.488061039 |
| CCDC77 | mRNA | 0.904509607 | PDRG1 | mRNA | 0.808820897 |
| CCDC93 | mRNA | 0.951058533 | PEX11G | mRNA | -1.117141715 |
| CCNA2 | mRNA | 3.494345133 | PGLYRP2 | mRNA | -1.54999018 |
| CCNB1 | mRNA | 3.276091248 | PGM1 | mRNA | -1.259921891 |
| CD109 | mRNA | 2.457053479 | PIPOX | mRNA | -0.971085474 |
| CDC37L1 | mRNA | -1.585748392 | PLG | mRNA | -1.705821672 |
| CDC7 | mRNA | 2.060770957 | PLIN1 | mRNA | -1.648717614 |
| CDKN2C | mRNA | 2.287694587 | PNPLA7 | mRNA | -1.375994097 |
| CENPF | mRNA | 3.886830228 | POGK | mRNA | 0.672197813 |
| CENPL | mRNA | 2.369053478 | PON1 | mRNA | -1.364585106 |
| CENPM | mRNA | 3.551795526 | PON3 | mRNA | -1.528895902 |
| CENPW | mRNA | 2.593919845 | PPM1G | mRNA | 0.582441445 |
| CEP41 | mRNA | 0.815275478 | PPP1R3B | mRNA | -1.667148972 |
| CHD1L | mRNA | 0.68363016 | PROC | mRNA | -0.80765273 |
| CHP1 | mRNA | -0.825575259 | PROZ | mRNA | -1.598523592 |
| CHRNE | mRNA | -0.796605624 | PRR11 | mRNA | 2.480831242 |
| CKAP5 | mRNA | 0.407920237 | PTPDC1 | mRNA | 0.899188066 |
| CLEC1B | mRNA | -5.193850222 | PTPRB | mRNA | -0.979690633 |
| CLU | mRNA | -0.927992047 | PZP | mRNA | -4.337896366 |
| CLYBL | mRNA | -0.920070942 | QDPR | mRNA | -1.077087114 |
| COBLL1 | mRNA | -1.098008079 | RAB10 | mRNA | 0.309957246 |
| COL25A1 | mRNA | -2.661524511 | RALA | mRNA | 0.354292517 |
| COLEC10 | mRNA | -3.935155835 | RAP2A | mRNA | 1.014583023 |
| COMMD2 | mRNA | 0.446560424 | RBMS3 | mRNA | -1.531635594 |
| CPEB3 | mRNA | -2.276348109 | RCAN1 | mRNA | -2.396381925 |
| CPN2 | mRNA | -1.104969858 | RCL1 | mRNA | -1.466885591 |
| CPSF3 | mRNA | 0.516582711 | RDH16 | mRNA | -2.016980219 |
| CPSF6 | mRNA | 0.521512884 | RHNO1 | mRNA | 1.143749389 |
| CRAT | mRNA | -0.691238023 | RIT1 | mRNA | 0.625995011 |
| CRHBP | mRNA | -4.452863068 | RMI1 | mRNA | 0.561283625 |
| CSNK1D | mRNA | 0.426913831 | RNF125 | mRNA | -1.885397831 |
| CSTF2 | mRNA | 0.872947247 | RNF2 | mRNA | 0.266958602 |
| CYB5D2 | mRNA | -0.858554668 | RRAGC | mRNA | 0.274283843 |
| CYP2C8 | mRNA | -2.680425905 | SAE1 | mRNA | 0.84115236 |
| CYP2C9 | mRNA | -1.822621056 | SARDH | mRNA | -1.4888929 |
| CYP4A11 | mRNA | -2.239195459 | SASS6 | mRNA | 1.03995332 |
| EED | mRNA | 0.571738895 | EFCAB7 | mRNA | 0.558114447 |
| UPF3B | mRNA | 0.879763414 | TTK | mRNA | 3.784646393 |
| UROC1 | mRNA | -2.288161282 | TTR | mRNA | -1.493684108 |
| USP1 | mRNA | 0.426126555 | UBAP2L | mRNA | 0.999297538 |
| VIPR1 | mRNA | -3.470966675 | UBE2Z | mRNA | 0.351875611 |
| WASF1 | mRNA | 0.649794758 | UPB1 | mRNA | -0.95447505 |
| WDHD1 | mRNA | 1.995542442 | FTCD | mRNA | -1.388702283 |
| XCR1 | mRNA | -1.053819018 | FTSJ3 | mRNA | 0.585638396 |
| XDH | mRNA | -1.283906298 | FUBP1 | mRNA | 0.36242058 |
| XPO5 | mRNA | 0.947407779 | FYN | mRNA | -0.951895217 |
| XPR1 | mRNA | 0.623423709 | GADD45G | mRNA | -1.370362106 |
| YEATS2 | mRNA | 1.039204357 | GALNT16 | mRNA | -0.927435211 |
| YWHAB | mRNA | 0.155619181 | GAS2L3 | mRNA | 1.152818393 |
| ZADH2 | mRNA | -1.065121183 | GCDH | mRNA | -1.298068505 |
| ZC2HC1C | mRNA | -1.414726999 | GFOD2 | mRNA | -0.572799008 |
| ZCCHC24 | mRNA | -1.104089079 | GGT5 | mRNA | -1.380078731 |
| ZIC2 | mRNA | 6.675185186 | GHR | mRNA | -2.470504198 |
| ZKSCAN4 | mRNA | 0.742230882 | GINS1 | mRNA | 2.734130867 |
| ZMYM1 | mRNA | 0.383251516 | GJB3 | mRNA | -1.555979984 |
| ZNF207 | mRNA | 0.474509583 | GLYCTK | mRNA | -0.663477457 |
| ZNF532 | mRNA | 0.950571776 | GNA14 | mRNA | -1.761009274 |
| ZNF544 | mRNA | 0.890878644 | SMIM14 | mRNA | -1.089659815 |
| ZNF605 | mRNA | 1.111511843 | SOCS2 | mRNA | -2.191368902 |
| ZRANB2 | mRNA | 0.386840702 | SORBS3 | mRNA | -0.75264403 |
| TK1 | mRNA | 2.323289596 | SPA17 | mRNA | 0.803561764 |
| TKT | mRNA | 1.506613595 | SPAG7 | mRNA | -0.427403071 |
| TMEM106C | mRNA | 1.496503838 | SPATA6L | mRNA | -0.905524344 |
| TMEM220 | mRNA | -1.496993564 | SPATS2 | mRNA | 1.569240458 |
| TPD52L2 | mRNA | 0.78626286 | SPNS1 | mRNA | 0.612692545 |
| TPMT | mRNA | -0.750017943 | SPRYD4 | mRNA | -1.317242161 |
| TPX2 | mRNA | 2.104402729 | SQSTM1 | mRNA | 1.069757281 |
| TRMT6 | mRNA | 0.628512081 | STARD5 | mRNA | -1.505967854 |
| TSPAN9 | mRNA | -0.653983364 | STEAP4 | mRNA | -1.658088129 |
| TTC36 | mRNA | -2.763675238 | STRN4 | mRNA | 0.575003433 |
| ENPEP | mRNA | -1.061170498 | STX6 | mRNA | 0.792988739 |
| EPAS1 | mRNA | -0.842898553 | SYNE2 | mRNA | -0.463424354 |
| EPHX2 | mRNA | -1.523848183 | TAGLN2 | mRNA | 0.879230211 |
| EPM2A | mRNA | -1.025914913 | TAPT1 | mRNA | -1.037965241 |
| ESR1 | mRNA | -2.471128716 | TBXA2R | mRNA | -1.789011906 |
| ETFDH | mRNA | -1.676097396 | TCF19 | mRNA | 2.774712915 |
| ETS2 | mRNA | -1.695272447 | TCF21 | mRNA | -1.724119475 |
| F2 | mRNA | -0.976903968 | TEK | mRNA | -1.372199628 |
| F9 | mRNA | -1.871819654 | EIF3B | mRNA | 0.612252683 |
| FABP5 | mRNA | 1.692225949 | ELFN1 | mRNA | -0.684742015 |
| FAM149A | mRNA | -1.36616063 | ENAH | mRNA | 1.553521676 |
| FAXDC2 | mRNA | -1.16484279 | CYP8B1 | mRNA | -1.505301325 |
| FBXO45 | mRNA | 0.4470575 | DBH | mRNA | -3.212252175 |
| FBXO8 | mRNA | -0.822298601 | DCAF11 | mRNA | -0.90246598 |
| FCGRT | mRNA | -0.849681842 | DCAF13 | mRNA | 1.045529363 |
| FEN1 | mRNA | 1.623886804 | DCAF16 | mRNA | 0.735499039 |
| FGD4 | mRNA | -0.947826702 | DCK | mRNA | 0.622954324 |
| FIGNL1 | mRNA | 1.092841534 | DCN | mRNA | -2.308426606 |
| SCGB3A1 | mRNA | -2.179685837 | DEPDC1 | mRNA | 3.758918772 |
| SCP2 | mRNA | -1.383862394 | DEPDC7 | mRNA | -1.235711522 |
| SELP | mRNA | -1.478372064 | DGAT2 | mRNA | -1.059919444 |
| SERPINA10 | mRNA | -1.160129225 | DHRS12 | mRNA | -0.693178622 |
| SERPINF2 | mRNA | -1.261556479 | DLAT | mRNA | 0.250674831 |
| SESTD1 | mRNA | 0.812560495 | DLG2 | mRNA | -1.149846104 |
| SHMT1 | mRNA | -1.278219071 | DMGDH | mRNA | -1.614715843 |
| SIRT3 | mRNA | -0.492935987 | DNASE1L3 | mRNA | -2.893476118 |
| SKA1 | mRNA | 4.474662242 | DROSHA | mRNA | 0.58922202 |
| SLC16A2 | mRNA | -1.180377765 | DTL | mRNA | 3.261429198 |
| SLC17A2 | mRNA | -0.925258478 | E2F3 | mRNA | 0.689197921 |
| SLC22A1 | mRNA | -2.367544573 | SLC46A3 | mRNA | -1.361024128 |
| SLC27A5 | mRNA | -1.516880384 | SLC7A2 | mRNA | -1.721450983 |
| SLC38A1 | mRNA | 1.003440255 | SLCO2B1 | mRNA | -0.98839177 |
| SLC38A4 | mRNA | -1.511886707 | SLCO4C1 | mRNA | -1.506554414 |
| SLC39A10 | mRNA | 1.264988365 | SMARCA2 | mRNA | -0.858444368 |
| SLC41A2 | mRNA | -1.37309225 | SMARCD1 | mRNA | 0.667418726 |
| CYP4F3 | mRNA | -0.894909848 | CYP4F12 | mRNA | -0.893293522 |
| CYP4V2 | mRNA | -1.436086783 |  |  |  |
